# Retraction Note: Post-COVID reactivation of latent *Bartonella henselae* infection: a case report and literature review

**DOI:** 10.1186/s12879-026-13036-9

**Published:** 2026-03-09

**Authors:** Yanzhao Dong, Ahmad Alhaskawi, Xiaodi Zou, Haiying Zhou, Sohaib Hasan Abdullah Ezzi, Vishnu Goutham Kota, Mohamed Hasan Abdulla Hasan Abdulla, Alenikova Olga, Sahar Ahmed Abdalbary, Hui Lu

**Affiliations:** 1 Department of Orthopedics, The First Affiliated Hospital, Zhejiang University, #79 Qingchun Road, Hangzhou, Zhejiang Province 310003 People’s Republic of China; 2 The Second Affiliated Hospital of Zhejiang Chinese Medical University, Hangzhou, Zhejiang Province 310003 People’s Republic of China; 3 School of Biomedical Science, Faculty of Medicine, The Chinese University of Hong Kong, Rm 706, 7/F, Lo Kwee-Seong Integrated Biomedical Science Bldg, Shatin, New Territories, Hong Kong; 4 Department of Orthopedics, Third Xiangya Hospital, Central South University, #138 Tongzi po Road, Changsha, Hunan Province 410013 People’s Republic of China; 5 Zhejiang University School of Medicine, #866 Yuhangtang Road, Hangzhou, Zhejiang Province 3100058 People’s Republic of China; 6 Department of Neurology, Republican Research and Clinical Center of Neurology and Neurosurgery, Skoriny, Minsk, Belarus; 7 Department of Orthopedic Physical Therapy, Faculty of Physical Therapy, Nahda University in Beni Suef, Beni Suef, Egypt

**Retraction Note: BMC Infectious Diseases (2024) 24:422**

10.1186/s12879-024-09336-7

The Editor has retracted this article. Concerns were raised regarding the availability of the NGS dataset. The authors were asked to deposit the NGS dataset in a public repository but have not done so. Therefore, the Editor has lost confidence in the conclusions presented in this article.

None of the authors have responded to correspondence regarding this retraction.

